# [^18^F]Fludarabine-PET in a murine model of multiple myeloma

**DOI:** 10.1371/journal.pone.0177125

**Published:** 2017-05-04

**Authors:** Narinée Hovhannisyan, Martine Dhilly, Martin Fidalgo, Fabien Fillesoye, Stéphane Guillouet, Brigitte Sola, Louisa Barré

**Affiliations:** 1 CEA, DRF/I2BM, LDM-TEP group, GIP Cyceron, Caen, France; 2 Normandie Univ, UNICAEN, CEA, CNRS, CHU Caen, ISTCT/LDM-TEP group, Caen, France; 3 Normandie Univ, INSERM UMR1245, UNICAEN, UNIROUEN, Caen, France; Wayne State University, UNITED STATES

## Abstract

**Purpose:**

Multiple myeloma (MM) is a haematological malignancy that affects plasma cells in the bone marrow. Recently, [^18^F]fludarabine has been introduced as an innovative PET radiotracer for imaging lymphoma. It demonstrated a great potential for accurate imaging of lymphoproliferative disorders. With the goal to question the usefulness of [^18^F]fludarabine-PET in other haematological diseases, an *in vivo* MM model was investigated.

**Methods:**

RPMI8226-GFP-Luc MM cells expressing the green fluorescent protein (GFP) as well as the luciferase reporter (Luc) were derived from the parental RPMI8226 cells. They were injected subcutaneously into the flank of *nude* mice. Myeloma tumour growth was followed using bioluminescence-based imaging (BLI) and characterised by immunohistochemistry (IHC). The tumour specificity of [^18^F]fludarabine was evaluated and compared to [^18^F]FDG.

**Results:**

The tumoural uptake of [^18^F]FDG was greater than that of [^18^F]fludarabine. However, the quantitative data extracted from IHC stainings were in better agreement with [^18^F]fludarabine, when compared to [^18^F]FDG. The relationship between the tumoural uptake of [^18^F]-labelled tracers and the BLI quantitative data was also in favour of [^18^F]fludarabine.

**Conclusion:**

Our results suggest that [^18^F]fludarabine-PET might represent an alternative and perhaps more specific modality for MM imaging when compared to [^18^F]FDG. Nevertheless, more investigations are required to extend this conclusion to humans.

## Introduction

MM is a haematological malignancy characterised by the accumulation of malignant plasma cells in the bone marrow, the presence of bone lytic lesions and the invasion of extra-medullary organs in later stages of the disease [1]. In the last decade, several imaging techniques such as MRI and [^18^F]FDG-PET/CT have emerged and were compared with conventional X-rays for the detection and monitoring of MM disease, allowing increased sensitivity and sensibility [2]. Nevertheless, [^18^F]FDG-PET/CT, which detects the metabolically active tumour cells and is useful for the diagnosis, staging and prognosis of MM, presents some limitations. In fact, in the cases of diffuse bone marrow infiltration or inflammatory lesions, its reduced sensitivity and specificity lead, respectively, to false-negatives and false-positives [3]. In order to circumvent those aspects and improve the efficacy of PET imaging for MM, there is an unmet need of a more accurate radiopharmaceutical. Recently, the early response to anti-myeloma therapy was evaluated in a mouse model with the radiolabelled amino acid *L*-methyl-[^11^C]-methionine ([^11^C]Met) which was revealed to be superior to [^18^F]FDG in terms of prediction of treatment efficacy [4]. However, the tumour specificity of [^11^C]Met remains to be established; without neglecting that ^11^C is a short half-life isotope and the radiopharmaceutical needs to be produced and evaluated on site.

[^18^F]Fludarabine, which is a novel PET probe, was developed for imaging B-cell lymphoma [5]. The specificity of [^18^F]fludarabine-PET was analyzed in various conditions and compared to [^18^F]FDG. We reported that, in xenograft models of follicular lymphoma, [^18^F]fludarabine displays a marked tumour *vs*. normal tissue contrast and has a high specificity even in tumours with necrotic and fibrotic components that arise following anticancer therapy [6,7]. We have also demonstrated the potential of [^18^F]fludarabine in distinguishing tumour from inflammatory tissue [8], which is a crucial capacity for predicting viable lymphoma in residual [^18^F]FDG avid masses after completion of therapy.

With the goal of extending those observations to other haematological diseases, [^18^F]fludarabine-PET was evaluated in a preclinical MM mouse model and compared to [^18^F]FDG.

## Materials & methods

### Ethics of experimentation

Experiments were conducted in accordance with the recommendations of European commission (86/609/EEC) and the French national committee for the care and the use of laboratory animals. Experiments were approved by the CENOMEXA (approvals N/09-11-12/32/11-15; N/17-11-12/40/11-15). Mice were housed under constant environmental conditions with 12/12 h light-dark cycles. Food and water were provided *ad libitum*. Their body weight remained constant throughout the investigation (35.12 ± 1.47 g). The behaviour of the mice during the experimental period was normal. Mice were maintained under isoflurane anesthesia throughout all procedures (induction 5%, maintenance 2%, with 70% N_2_O/30% O_2_; Minerve system, Bioscan, France). Body temperature was maintained at 37°C using a feedback controlled system (Minerve, Bioscan, France) during the imaging sessions. Mice were euthanized by cervical dislocation under deep anesthesia (5%) at the end of imaging studies.

### Cell cultures and *in vitro* analysis of luciferase activity

RPMI8226-GFP-Luc cells derived from the parental RPMI8226 MM cell line were genetically engineered to express the green fluorescent protein (GFP) and the luciferase gene for *in-vivo* examinations with BLI [9]. They were maintained in RPMI 1640 medium (Lonza, France) containing 10% fetal calf serum (PAA laboratories, France), 2 mM L-glutamine, and antibiotics (Lonza, France). RPMI8226-GFP-Luc cells were seeded in 24-well plates in complete medium at various densities (10^5^ to 10^7^ cells/mL) and 125 μg/mL D-Luciferin (Promega, France) was added to each well. Plates were incubated for 5 min at 37°C. Bioluminescent signals produced by MM cells were captured and imaged by a PhotonIMAGER and quantified with the M3Vision software (Biospace-Lab, France). Another type of human myeloma cell line LP-1 was also investigated in similar cell culture conditions (without expression of GFP) for the in-vitro evaluation of [^18^F]fludarabine uptake.

### Cellular uptake of [^18^F]fludarabine

RPMI8226-GFP-Luc and LP-1 cells were maintained as described previously. Cells (10^5^, 5x10^6^ and 10^7^ cells/1.5 mL) were incubated with [^18^F]fludarabine (0.9 MBq/mL, 0.01 μg; produced in-house) at 37° for 60 min in serum free RPMI 1640 medium. Cells were further washed twice with PBS and harvested. Cell viability was assessed by trypan blue exclusion test. The radioactivity in the cell pellets was counted with an automatic well-type gamma counter (Cobra, PerkinElmer, France) and the data were expressed as a percentage of incorporated [^18^F]fludarabine. A competition study with a _~_1000-fold excess of non-radioactive fludarabine (10 μg) was performed to reaffirm the similarity of the [^18^F]fludarabine transportation and retention pathway when compared to fludarabine drug.

### Animal model and tumour phenotyping

A series of ten six-week old *nude* mice were injected subcutaneously into the flank with 2 x 10^6^ RPMI8226-GFP-Luc cells half-mixed in Matrigel (BD Bioscience, France). BLI was used to assess the growth of MM cells twice a week. Five min after intraperitoneal injection of 75 mg/kg D-luciferine, bioluminescent signals were acquired for 5 min with the PhotonIMAGER. Total body luminescence was calculated using the M3Vision software. At the end of the experiment, the mice were sacrificed and the tumours were excised and immediately fixed in paraformaldehyde (4%) prior to dehydration and embedded in paraffin. Five micron-thick sections were stained with haematoxylin and eosin (HES) for histological examination. For IHC, the sections were labelled with the following primary antibodies: mouse anti-CD138 (M7228, Dako, Denmark) as a marker of MM cells; rabbit anti-phospho(p)-H3 (#9701, Cell Signaling Technology, USA) as a marker of proliferation; rat anti-CD34 (HM1015, HyCult Biotechnology, Netherlands) as a marker of endothelial cells. The sections were then processed with the N-Histofine^®^ Simple Stain mouse kit (414151F, Nichirei Biosciences INC, Japan). Immunostainings were revealed with an appropriate diaminobenzidine kit (Thermo Fischer Scientific, USA). All slides were immunostained the same day, guaranteeing a standardized intensity of staining, and visualized with Axiophot microscope (Carl-Zeiss, Deutschland).

### Imaging sessions

A second series of five *nude* mice were engrafted with MM cells as described above. The tumoural growth was monitored by bioluminescence approach which is also described in the previous section. PET/CT imaging was performed with an Inveon microPET/CT scanner (Siemens, USA), with [^18^F]fludarabine on the last BLI acquisition day (23) and with [^18^F]FDG the day after, on the same mice. The information on the in-house radiosynthesis of [^18^F]fludarabine can be found elsewhere (S1 Appendix) [5]; the [^18^F]FDG was purchased from Cyclopharma S.A (France). PET acquisition was performed after intravenous (caudal vein) injection of [^18^F]fludarabine (10.51 ± 0.70 MBq, n = 5) or [^18^F]FDG (11.74 ± 0.18 MBq, n = 4 [1 died]). Time-activity curves (TACs) were obtained for three mice with the dynamic imaging up to 90 min post-injection. The static scans were acquired 40 to 60 min after radiotracer administration; since the tumoural uptake had rapidly reached a relative plateau with both radiotracers in the present animal model. The applied acquisition and reconstruction parameters for the PET scans are available in our former publication [7]. Briefly, the emission scan was acquired with default settings of coincidence timing window of 3.4 ns and an energy window of 350 to 650 keV. PET images were reconstructed with 3-dimensional maximum *a posteriori* (OSEM3D/MAP) reconstruction algorithm. The data were reconstructed into 128 x 128 x 159 matrix images. Data were normalized and dead-time, random, scatter as well as attenuation correction (based on CT) were applied. At the end of imaging investigations, the tumours were processed for IHC examinations; in addition to above mentioned stainings, F4/80 was used as a marker of inflammatory cells. A simple ranking system (o: no staining, from + to +++) was applied to depict the various degrees of staining intensity.

### Analysis of multimodal tumour data

Ventral plus dorsal BLIs were determined by quantifying photon flux inside a circle region of interest, encompassing the tumour.

To obtain the tumoural uptake of PET radiotracers, volumes of interests were manually defined on the coronal section of PET scan (PMOD 3.6, PMOD-Technologies, Switzerland); co-registered CT scan was used for the adjustment of the delineation. Radioactivity quantification was based on the pixel with the highest uptake (SUV_max_) and on the region encompassed with an automated irregular 3D-isocontour (SUV_mean_).

### Statistical analyses

The correlation- and t-tests were performed with Prism 4.03 software (GraphPad, USA); *p*-value < 0.05 was considered to be significant.

## Results

To question the usefulness of the [^18^F]fludarabine in MM imaging, we used two types of human myeloma cell lines the RPMI8226-GFP-Luc able to graft into immunodeficient mice and to progress as myeloma tumour [9] and the LP-1 for *in-vitro* use only. We first established that the cellular uptake of [^18^F]fludarabine was similar in both RPMI8226-GFP-Luc and LP-1 cells (S1 Fig). The competition study with an excess of non-radioactive fludarabine revealed a 2.5-fold decrease in cell-associated [^18^F]fludarabine uptake, in both groups of MM cells. For further examinations, we continued to consider RPMI8226-GFP-Luc only, which was derived from the parental RPMI8226 MM cell line, with no modification of biological characteristics—cell doubling time of 25 h, similar distribution of cells within the cell cycle [10].

**Fig 1:**
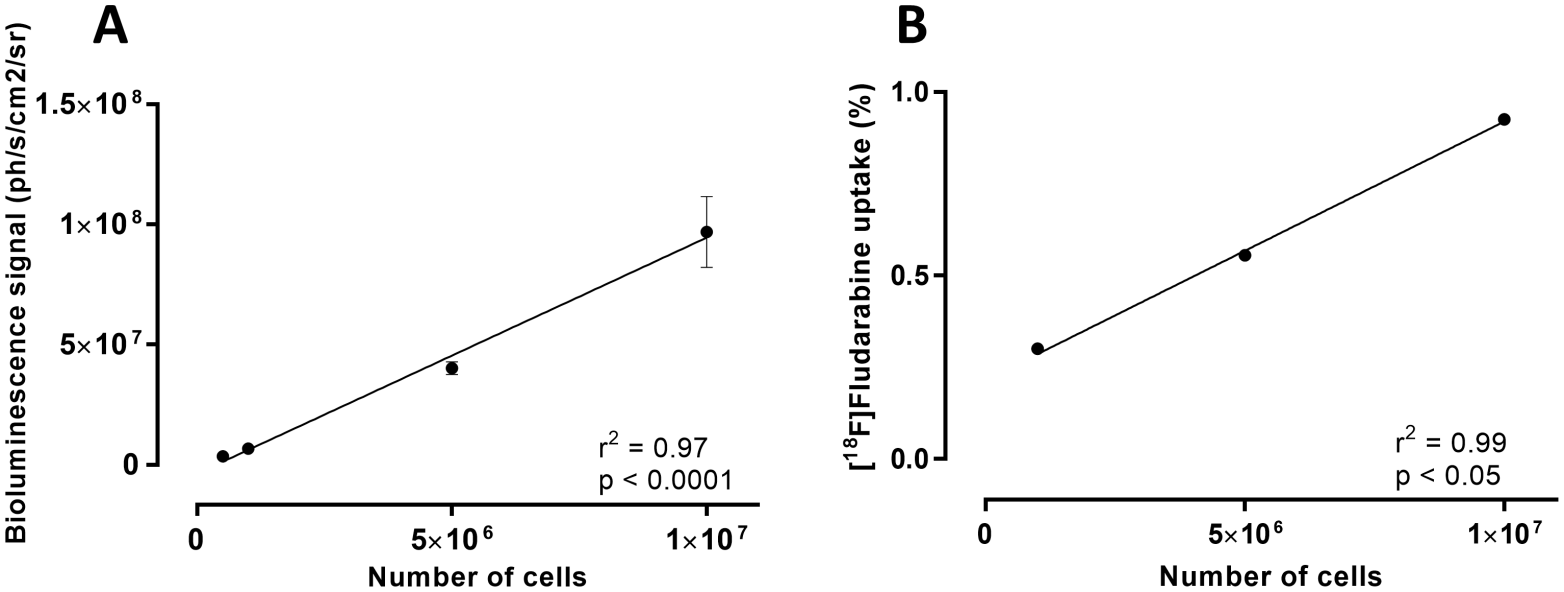
*In vitro* analysis of BLI and [^18^F]fludarabine signals. Relationship between the number of RPMI8226-GFP-Luc cells and (A) BLI intensity, (B) [^18^F]fludarabine uptake. Error bar: mean ± SD, in triplicate.

In an *in vitro* setting, we observed that BLI signal, as well as the [^18^F]fludarabine uptake, were strictly proportional (r^2^ = 0.97, p < 0.0001 and r^2^ = 0.99, p < 0.05, respectively) to the number of viable and metabolically active cells (Fig 1).

**Fig 2:**
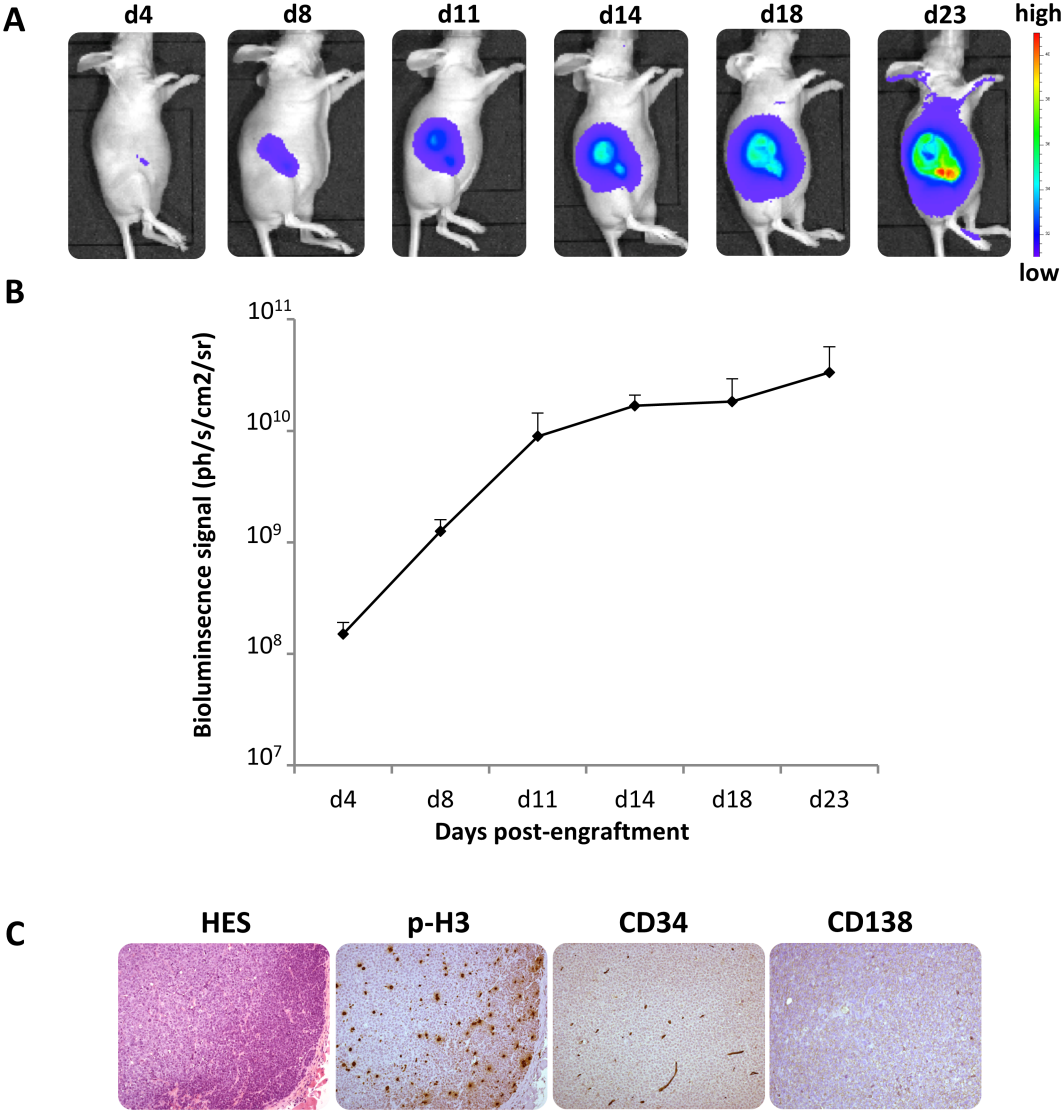
MM mouse model. (A) Sequential BLI scans of a mouse injected with 2 x 10^6^ RPMI 8226-GFP-Luc cells into the flank. (B) Luciferase activity quantified at the same time points for ten mice, mean ± SD. (C) Representative immunohistochemistry images (x100 magnification) of tumour sections of the mouse, the BLI scans of which are displayed above. HES: nuclear stain, CD138: identity of the cancer cells-of-origin, p-H3: reflects proliferation, CD34: reflects neoangiogenesis.

Then, a series of ten *nude* mice were injected with 2 x 10^6^ RPMI8226-GFP-Luc cells into the flank. The tumoural growth was monitored regularly (from day 4 to 23) by non-invasive BLI and the luciferase activity was quantified (Fig 2A and 2B). In the terminal stage of cell growth (day 23), the mice were sacrificed and the identity of tumoural cells was checked by morphological analysis (HES staining) and immunostaining [Fig 2C]. HES and CD138 stainings confirmed the presence of myeloma cells that are proliferating (p-H3-positive). Neoangiogenesis occurred at the site of tumour engraftment as deduced from the CD34 staining. These observations validated the mouse model.

**Fig 3:**
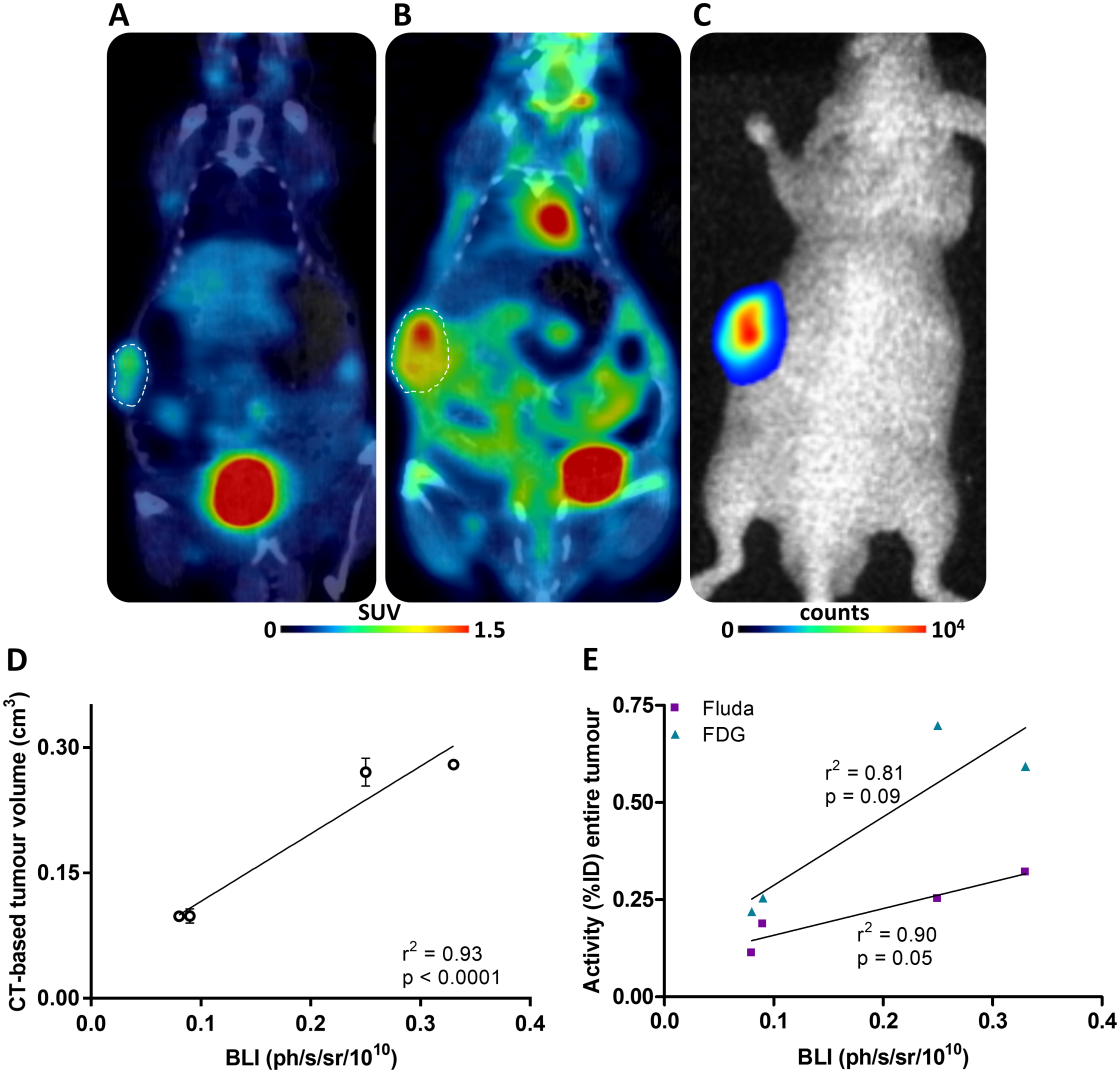
Combined PET/CT and BLI data. (A) Representative [^18^F]fludarabine- and (B) [^18^F]FDG-PET/CT fused scans (40–60 min post-injection), as well as (C) the corresponding BLI scan from the same mouse. (D) Relationship between CT-based tumour volume (mean ± SD, in duplicate) and BLI intensity. (E) Relationship between [^18^F]-labelled tracers uptake and BLI intensity.

A second series of engrafted mice (n = 5) was used for the multimodality imaging sessions. A representative illustration of paired [^18^F]fludarabine- and [^18^F]FDG-PET/CT scans as well as of BLI image of the same mouse can be found in Fig 3A–3C. A strong correlation (r^2^ = 0.93, p < 0.0001) was observed between the BLI signal and the tumour volume assessed by the CT scans (Fig 3D). Correlations were also established between the tumoural uptake of above-mentioned [^18^F]-labelled tracers and the BLI-integrated intensity, which was in favour of [^18^F]fludarabine-PET (r^2^ = 0.90, p = 0.05) (Fig 3E).

**Fig 4:**
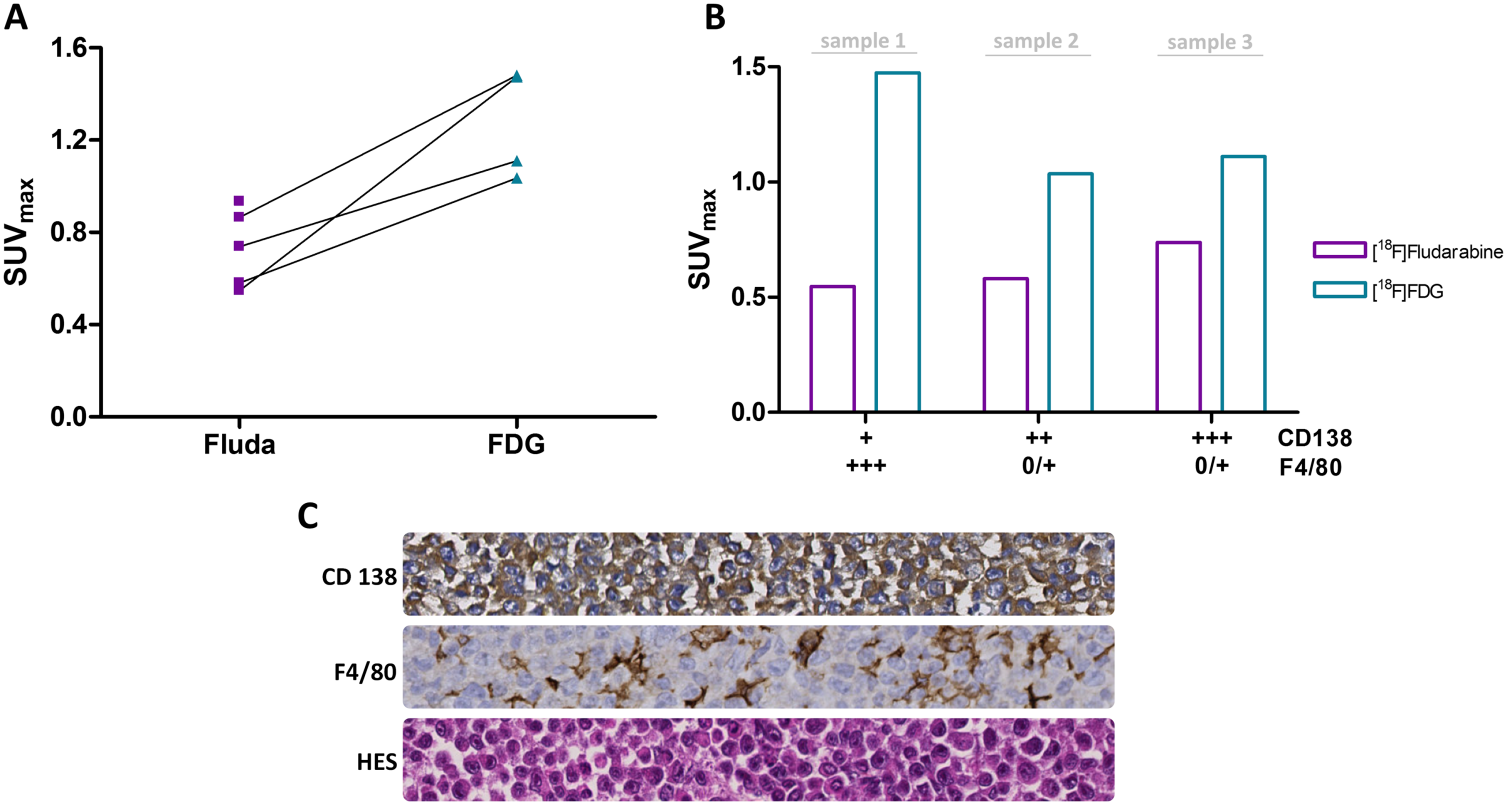
PET analysis and histology. (A) Uptake in tumour for [^18^F]fludarabine and [^18^F]FDG (40–60 min post-injection), paired samples are connected with lines. (B) Combined quantitative values of PET and IHC. (C) Histological illustrations with x400 microscopic views—staining of adjacent sections—of an MM tumour. HES: nuclear stain, CD138: identity of the cancer cells of origin, F4/80: marker of inflammatory cells.

TACs obtained with [^18^F]fludarabine for tumour and muscle (non-target tissue) showed a greater clearance from normal tissue when compared to tumoural masses in which the uptake rapidly reached a relative plateau, with a tumour to muscle ratio of 2.08 ± 0.42 and 2.86 ± 0.72 (n = 3) at 60 and 90 min post-injection, respectively (S2 Fig). Although in the present MM model the tumoural uptake of [^18^F]FDG [SUV_max_ = 1.27 ± 0.23, with 0.44 ± 0.06 in muscle, n = 4] tended to be greater than that of [^18^F]fludarabine [SUV_max_ = 0.73 ± 0.17, with 0.42 ± 0.09 in muscle, n = 5] (Fig 4A), the IHC revelations based on CD138 and F4/80 stainings were in better agreement with [^18^F]fludarabine-PET (Fig 4B). Indeed, in contrast to the glucose analogue, the concentration of [^18^F]fludarabine activity was greater in tumours with higher level of CD138. The presence of inflammatory cells, revealed by F4/80 staining, did not affect the tracer uptake. As for [^18^F]FDG-PET, the greatest SUV_max_ was observed for the sample with the lowest CD138 and highest F4/80 levels (similar results were observed with SUV_mean_, data not shown).

## Discussions

Recently, [^18^F]fludarabine has been introduced as an innovative PET radiotracer for imaging lymphoma, which demonstrates great potential for accurate imaging of lymphoproliferative disorders. This radiotracer has already been questioned in several investigations [6–8], suggesting that [^18^F]fludarabine imaging provides tumour-specific information and in various conditions may be more informative than [^18^F]FDG. In summary, the former preclinical studies revealed elevated [^18^F]fludarabine tumor/background ratio and capability of this tracer to detect residual active masses following chemotherapy treatment in xenograft models of follicular lymphoma, as well as negligible uptake in a non-bacterial model of inflammation. Furthermore, in our first-in-human study, [^18^F]fludarabine demonstrated lack of uptake in several FDG-avid mediastinal sites (DLBCL patient), which were confirmed to be non-pathological lymph nodes [11]. To expand the scope of this novel PET probe for B-cell lymphoma imaging, [^18^F]fludarabine was evaluated in a xenograft MM murine model. MM is characterised by malignant proliferation of plasma cells, predominantly localized in the bone marrow. Furthermore, extra-medullary disease may also be present and, thus, PET based on [^18^F]FDG is a more sensitive imaging technique when compared to conventional radiography or MRI [12]. However, the role of [^18^F]FDG-PET in the management of MM remains limited because of its lack of sensitivity for detecting diffuse bone marrow involvement, small skull lesions due to the physiological [^18^F]FDG uptake in brain [1].

The goal of this study was to evaluate the feasibility of [^18^F]fludarabine in PET imaging of MM. For this, two human myeloma cell lines were investigated: the RPMI8226-GFP-Luc able to graft into immunodeficient mice and to progress as myeloma tumour [9] and the LP-1 for *in-vitro* use only. We demonstrated that the [^18^F]fludarabine was trapped in both groups of cells with similar cell-associated activity, which would indicate the potential of this radiotracer to image MM. Moreover, in the presence of an excess of non-radioactive fludarabine, the cellular uptake of [^18^F]fludarabine was dropped significantly. This result suggests that both [^18^F]fludarabine and fludarabine have a similar mechanism of transportation and retention into the cells and the labelling of the drug did not affect its highly specific targeting mechanism for tumoural cells [13]. Our further examinations were based on RPMI8226-GFP-Luc cell line. In an *in vitro* setting, we observed that BLI signal was strictly proportional to the number of viable and metabolically active cells, hence, the tumoural growth was monitored with bioluminescence approach in our further *in-vivo* study. [^18^F]Fludarabine uptake was also significantly related to the number of cells suggesting the robustness of the transportation and trapping mechanisms of this probe. The MM mouse model was then validated in a series of *nude* mice by injection of 2 x 10^6^ RPMI8226-GFP-Luc cells into the flank, and in agreement with previous data [10], *bona fide* myeloma tumours grew at the site of injection as validated by BLI and IHC analyses. A second series of engrafted mice was used for the multimodality imaging sessions. A linear relationship was observed between the tumour volume assessed by CT and the BLI signal. [^18^F]Fludarabine-PET demonstrated slightly stronger correlation than [^18^F]FDG with regard to their tumoural uptake when plotted as a function of BLI-integrated intensity. Dynamic PET acquisitions with [^18^F]fludarabine showed progressive clearance from physiological tissues, while retention in tumoural masses. The IHC analysis based on CD138 and F4/80 stainings revealed that [^18^F]fludarabine activity was greater in tumours with higher level of CD138, while that of [^18^F]FDG was elevated for the sample with the lowest CD138 and highest F4/80 levels. Indeed, the increased non-specific accumulation of [^18^F]FDG in inflammatory cells is a well-known biological phenomenon. A comparative intertracer study between [^18^F]fludarabine- and [^18^F]FDG-PET has previously been investigated in a murine model, revealing significantly higher [^18^F]FDG uptake in both early and late stages of inflammation [8].

Our data suggested that [^18^F]fludarabine-PET might represent an alternative and perhaps more specific modality for MM imaging when compared to [^18^F]FDG-PET. Nevertheless, more investigations are required to further elucidate the role of [^18^F]fludarabine-PET in MM and to extend this conclusion to humans.

## Supporting information

S1 Appendix Radiosynthesis of [^18^F]Fludarabine. (PDF)

S1 Fig *In vitro* cellular uptake of [^18^F]fludarabine. Cell-associated activity of [^18^F]fludarabine in RPMI8226-GFP-Luc and LP-1 human myeloma cell lines (10^7^ cells/1.5 mL) at 1h incubation (37°C). Cmpt: uptake of [^18^F]fludarabine in competition condition with a _~_1000-fold excess of non-radioactive fludarabine. Error bar: mean ± SD, in triplicate.
(TIF)

S2 Fig Time-activity curves with [^18^F]fludarabine. Data obtained from dynamic PET scans for tumour (bold lines, a colour per mouse) and muscle, as a non-target tissue (dotted lines).
(TIF)
