# Supplementary material for: [18F]Fludarabine-PET in a murine model of multiple myeloma
Source: PLoS One. 2017 May 4;12(5):e0177125. doi: 10.1371/journal.pone.0177125 (PMC5417674; doi:10.1371/journal.pone.0177125)
Supplement: S1 Appendix — (PDF) [file pone.0177125.s001.pdf]

Radiosynthesis of [^{18}F]fludarabine

The radiolabeling of [^{18}F]fludarabine was based on the use of a nitro group, at the 2-position of the purine moiety of the protected starting material, for the nucleophilic aromatic substitution by reaction with [^{18}F]fluoride. [^{18}F]Fluoride was produced *via* an ^{18}O(p,n)^{18}F nuclear reaction in a cyclotron Cyclone® 18/9 IBA (IBA, Louvain la Neuve, Belgium). A full computerized, one pot/two step ^{18}F-Fludarabine-synthesis method (fluorination and deprotection, S1 Appendix Fig. 1) was developed on the TRACERlab® FX-FN module (GE Healthcare, Buc, France) for routine production [1]. [^{18}F]fludarabine was produced in 48±3% yield (decay corrected at the end of bombardment) with a radiochemical purity > 99% and a specific activity of 310±72 GBq/µmol after 85 min of synthesis including HPLC purification (Waters µBondapak C18, 10 µm, 7.8x300 mm; mobile phase water/ethanol 98:2; flow rate 4 mL/min; λ=254 nm; *tr*=18 min). The tests for quality control as chemical and radiochemical purity (S1 Appendix Fig. 2), residual solvents, visual inspection, pH were in accordance to the European Pharmacopoeia specifications [2]. The stability was established for 8h.

S1 Appendix Fig. 1: Radiosynthesis of [^{18}F]fludarabine.

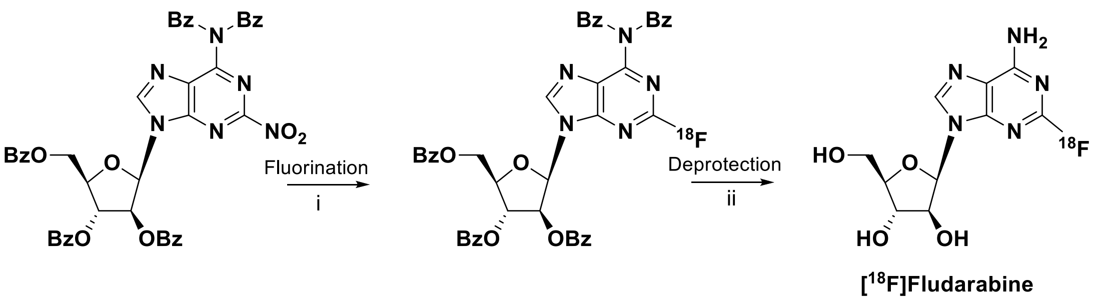

i) [18F]KF/K222/K2CO3, CH3CN, 55-60°C, 7min ii) MeOH/NH3.H20 70°C, 20min

S1 Appendix Fig. 2: Analytical HPLC chromatogram of [^{18}F]fludarabine: Reverse phase HPLC - Nucleosil 50-5 C18 ec 250x4 mm Macherey Nagel; mobile phase: water/acetonitrile 94:6; flow rate: 1 mL/min; λ=254 nm.

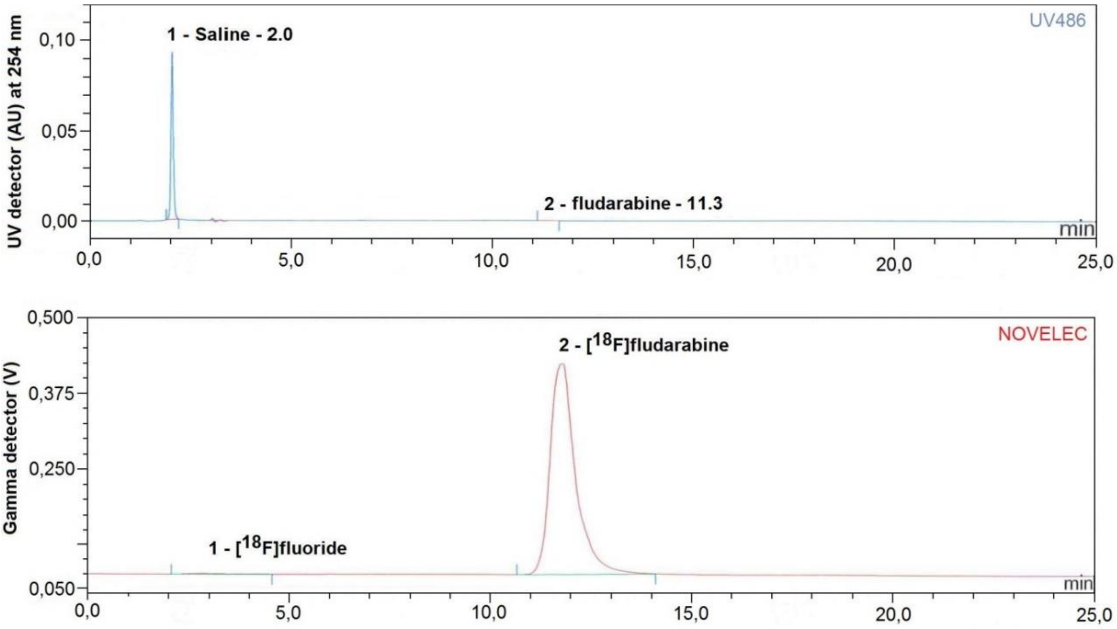

No. NOVELEC	Peak Name NOVELEC	Ret. Time (detected) NOVELEC min	Area NOVELEC V*min	Rel. Area NOVELEC %	Rel. Area % corrected %
1	[^{18}F]fluoride	2,83	0,001207	0,47	0,35
2	[^{18}F]fludarabine	11,77	0,255061	99,53	99,65

1. Guillouet S, Patin D, Tirel O, Delamare J, Gourand F, Deloye JB, et al. Fully automated radiosynthesis of 2-[18F]fludarabine for PET imaging of low-grade lymphoma. Mol Imaging Biol. 2014; 16: 28-35.
2. Verbruggen A, Coenen HH, Deverre JR, Guilloteau D, Langstrom B, Salvadori PA, *et al*. Guideline to regulations for radiopharmaceuticals in early phase clinical trials in the EU. Eur J Nucl Med Mol Imaging 2008; 35: 2144–2151.
