# Supplementary figures and images for: [18F]Fludarabine-PET in a murine model of multiple myeloma
Source: PLoS One. 2017 May 4;12(5):e0177125. doi: 10.1371/journal.pone.0177125 (PMC5417674; doi:10.1371/journal.pone.0177125)

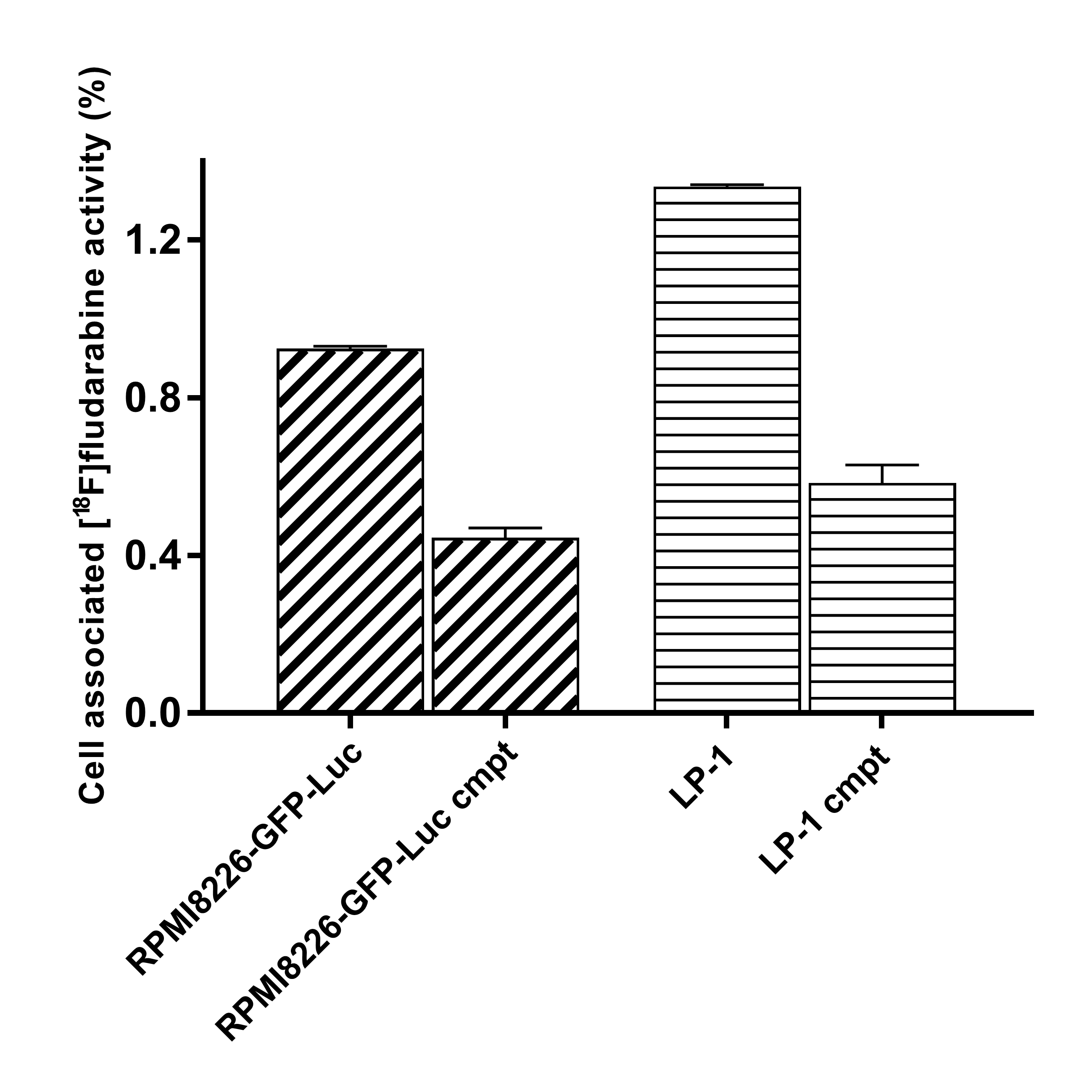

Supplement: S1 Fig — Cell-associated activity of [18F]fludarabine in RPMI8226-GFP-Luc and LP-1 human myeloma cell lines (107 cells/1.5 mL) at 1h incubation (37°C). Cmpt: uptake of [18F]fludarabine in competition condition with a ~1000-fold excess of non-radioactive fludarabine. Error bar: mean ± SD, in triplicate. (TIF) [file pone.0177125.s002.tif]

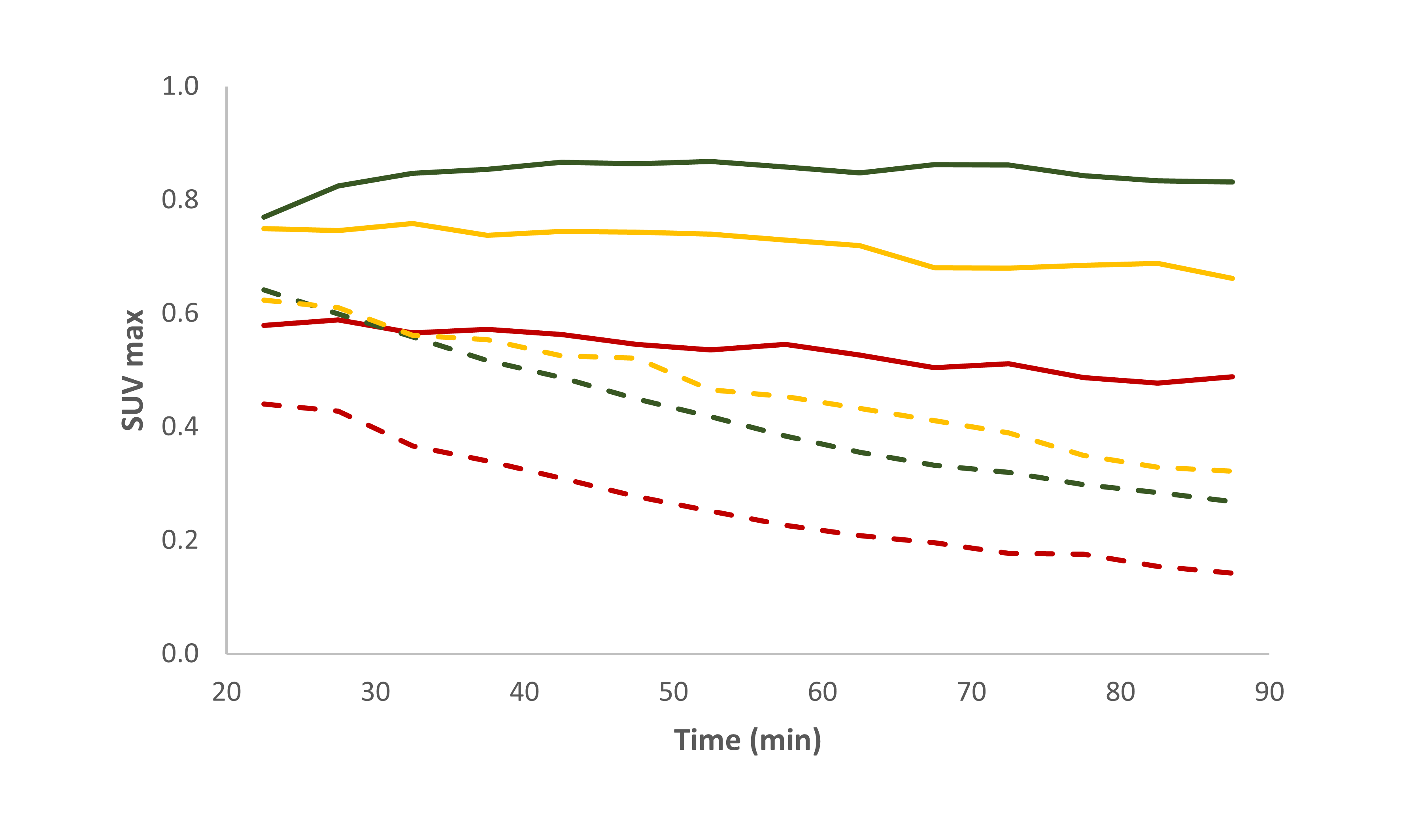

Supplement: S2 Fig — Data obtained from dynamic PET scans for tumour (bold lines, a colour per mouse) and muscle, as a non-target tissue (dotted lines). (TIF) [file pone.0177125.s003.tif]
